# Effect of PI3K/AKT/mTOR signaling pathway-based clustered nursing care combined with papaverine injection on vascular inflammation and vascular crisis after replantation of severed fingers

**DOI:** 10.1007/s11010-023-04796-y

**Published:** 2023-07-25

**Authors:** Na Wang, Haijing Xiao, Hongyan Lu, Kai Chen, Shuhong Zhang, Fei Liu, Ning Zhang, Haijing Zhang, Siyu Chen, Xiaoli Xu

**Affiliations:** 1 Department of Hand, Foot and Ankle Surgery, General Hospital of Ningxia Medical University, Yinchuan, China; 2 Outpatient Department of the People’s Hospital of Ningxia Hui Autonomous Region, Yinchuan, China; 3 Nursing Department, General Hospital of Ningxia Medical University, 804 Shengli South Street, Xingqing District, Yinchuan, 750004 Ningxia China; 4 Department of Hepatobiliary Surgery, General Hospital of Ningxia Medical University, Yinchuan, China; 5 Department of Stomatology, General Hospital of Ningxia Medical University, Yinchuan, China

**Keywords:** Severed finger replantation, PI3K/AKT/mTOR signaling pathway, Papaverine injection, Clustered nursing care, Vascular inflammation, Vascular crisis

## Abstract

This research aimed to investigate the effect of PI3K (phosphatidylinositol 3-kinase)/AKT (protein kinase B)/mTOR (mammalian target protein of rapamycin) signaling pathway-based clustering care combined with papaverine injection on vascular inflammation and vascular crisis after finger amputation and replantation. 100 patients admitted in General Hospital of Ningxia Medical University from April 2022 to December 2022 for replantation of severed fingers were selected and divided into a control group (*n* = 50) and an observation group (*n* = 50) using the randomized grouping principle. The control group received a papaverine injection and general nursing care, the observation group received a papaverine injection and clustered care. The pain score; constipation incidence; replantation finger survival rate; physician, nurse, and patient satisfaction; serum inflammatory factors; vascular crisis parameters; and occurrence of adverse reactions were compared between the two patient groups. Enzyme-linked immunosorbent assay was performed to detect PI3K, AKT, and mTOR protein concentrations in the venous blood of the two groups, and statistical analysis of the data was performed. On postoperative day 7, the pain score and incidence of constipation in the observation group were lower than those in the control group (*P* < 0.05); the survival rate of reimplanted fingers in the observation group was 88.00%, which was higher than that in the control group 80.00% (*P* < 0.05); the satisfaction of doctors, nurses, and patients in the observation group was higher than that in the control group; the concentrations of interleukin-1 (IL-1), tumor necrosis factor (TNF-α), blood flow resistance index (RI), and arterial pulsatility index (PI) in the observation group were lower than those in the control group, while the concentration of interleukin-10 (IL-10), vascular diameter, and Vm (mean blood flow velocity) were higher in the observation group than those in the control group; the differences were statistically significant (*P* < 0.05). The difference in the incidence of adverse reactions between the two groups was not statistically significant (*P* > 0.05). The concentrations of PI3K, AKT, and mTOR proteins in the observation group were higher than those in the control group (*P* < 0.05). The concentrations of PI3K, AKT, and mTOR proteins in the observation group were higher than those in the control group (*P* < 0.05). Overall, these findings suggest that clustered care combined with papaverine injection reduces vascular inflammatory symptoms and vascular crisis in the treatment of severed finger replantation through the PI3K/AKT/mTOR signaling pathway.

**Supplementary Information:**

The online version contains supplementary material available at 10.1007/s11010-023-04796-y.

## Introduction

With the widespread use of industrial instruments and the rapid development of public transportation, the incidence of finger amputation due to industrial and traffic accidents has increased. The survival rate of severed fingers after replantation can be effectively improved by comprehensive treatment, including replantation surgery and postoperative medication, psychotherapy, and rehabilitation [1]. Through surgery, blood vessels, nerves, and other tissues can be anastomosed to restore blood supply so that the tissue continues to maintain biological activity and physiological function. However, due to the delicate blood vessels of the finger, the surgical operation requires more delicacy and requires a longer period of bed rest after surgery, which can easily lead to critical conditions such as vasospasm, which can significantly affect the blood supply of the severed finger, even cause necrosis and detachment of the severed finger [2, 3]. Some studies have shown that the incidence of vascular crisis after replantation remains at 10.00%–20.00% and seriously affects the survival of reimplanted fingers [4, 5]. Therefore, the prevention of vascular crisis after replantation of severed fingers is crucial and urgently requires scientific and effective treatment methods or care intervention models.

Papaverine bases belong to opioid alkaloids, which can induce smooth muscle relaxation, relieve pain and limb anemia symptoms, promote blood flow to the diseased area, and prevent vasospasms; however, papaverine bases can induce vascular inflammation, whether taken intramuscularly or through micropump injection [6]. As a commonly used vasodilator, papaverine inhibits phosphodiesterase activity in vascular smooth muscle cells and calcium inward flow across vascular smooth muscle cell membranes, dilating peripheral vasculature, organ vessels, and bronchial and digestive smooth muscles,it has thus been widely used in the prevention and treatment of vasospasm after finger replantation [7]. With the introduction of a new method for papaverine injection, many side effects of papaverine have decreased, but papaverine-induced vascular inflammation is still common [8]. It has been found that interleukin-10 (IL-10) is an effective biomarker of an anti-inflammatory response,it is an inhibitor of neutrophil activation by pro-inflammatory factors, resulting in the inability of bacteria in lysozymes to be released, safeguarding the integrity of the vascular lining, and therefore not triggering an inflammatory response [9].

Clustered care is a new model of nursing intervention that brings together a range of evidence-based treatment and care interventions to manage patients with a difficult-to-treat clinical condition [10]. It was first proposed by the Institute for the Advancement of Research in the United States, with the aim of providing quality health care services and outcomes for patients [11]. This model of care is based on the development of 3–6 evidence-based goals of care, measures that must be agreed upon in clinical practice, implemented at the same time and in the same place, and each be judged as “yes” or “no.” The combined implementation of these measures is likely to result in better outcomes than their individual implementation. In recent foreign studies, clustered care has been shown to reduce surgical infection rates [12], chronic obstructive pulmonary disease [13], decompensated cirrhosis [14], and hip fractures [15].

The PI3K (phosphatidylinositol 3-kinase)/AKT (protein kinase B)/mTOR (mammalian target of rapamycin protein) signaling pathway is an important metabolic information transduction pathway involved in a variety of physiological and pathological activities in vivo, and several studies have shown that trauma, surgery, and inflammation can activate the PI3K/AKT/mTOR pathway in tissue cells [16, 17]. It is particularly important to prevent the stimulation of coagulation through endothelial cell injury mechanisms and promote healing at the anastomotic end of vessels during the recanalization of injured arteries after finger amputation surgery. The PI3K/AKT/mTOR pathway directly regulates the morphology, proliferation, survival, and function of endothelial cells, participates in endothelial cytoskeleton remodeling, and promotes endothelial repair, while indirectly regulating vascular endothelial growth factor (VEGF) through the positive regulation of hypoxia inducible factor-1α (HIF-1α). The enhanced release of various chemokines also indirectly regulates vascular endothelial cell proliferation and growth, vasodilation, and vascular neovascularization [18, 19].

Preliminary studies have been conducted on the use of centralized care in replantation of severed fingers [20, 21] and found that it reduces the pain and improves the comfort and sleep quality of patients after replantation. However, no systematic studies have been reported on how centralized care affects vascular inflammation and vascular crisis. The present study aimed to investigate the effect and mechanism of action of a clustered nursing intervention strategy combined with papaverine injection in patients who had undergone severed finger replantation to reduce the incidence of vascular crisis and improve the survival rate of reimplanted fingers by avoiding risk factors. The study also aimed to provide a scientific basis for healthcare professionals for preventing a vascular crisis after replantation.

## Methods

### General information

**Fig. 1:**
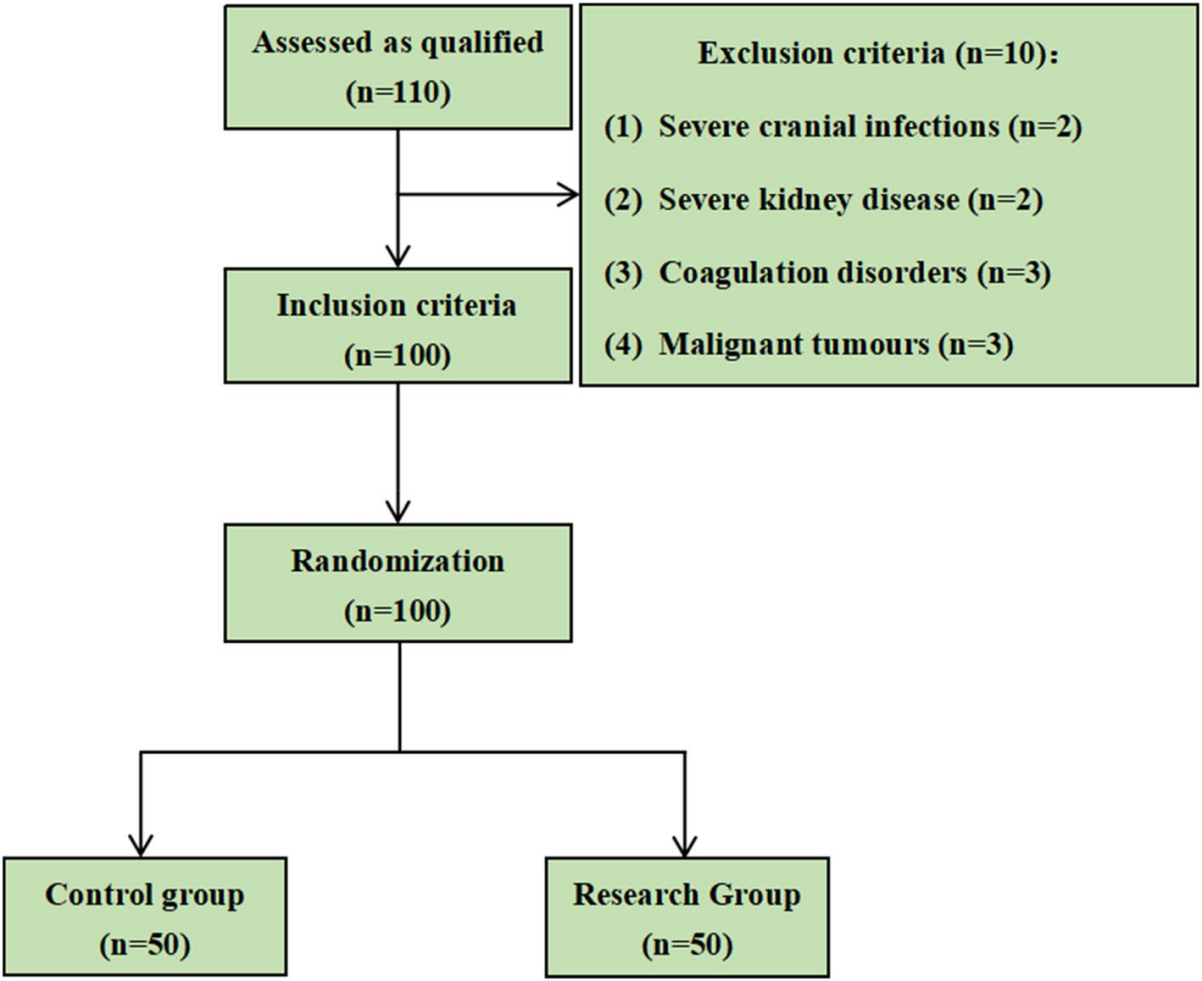
The specific flow chart for the patient selection and treatment process

The sample for the present study included hundred patients who were admitted in General Hospital of Ningxia Medical University from April 2022 to December 2022 for replantation of severed fingers. The patients were divided into a control group (*n* = 50) and an observation group (*n* = 50) according to the randomized grouping principle. The control group received papaverine injection treatment and general care, while the observation group received papaverine injection treatment and clustered care. The specific flow chart for the patient selection and treatment process is shown in Fig. 1. The study was approved by the ethics committee of the General Hospital of Ningxia Medical University (Approval No.: KYLL-2022-0361), and patients gave written informed consent for participation in the study by signing the informed consent form.

The inclusion criteria were as follows: time from injury to hospital admission ≤ 6 h, toleration of papaverine base treatment, American Society of Anesthesiologists classification [22] grade I–II.

The exclusion criteria were as follows: pregnancy, lactation, malignancy, hepatic or renal insufficiency, presence of severe cranial infection, and coagulation disorders.

### Methods

#### Replantation of broken fingers

**Fig. 2:**
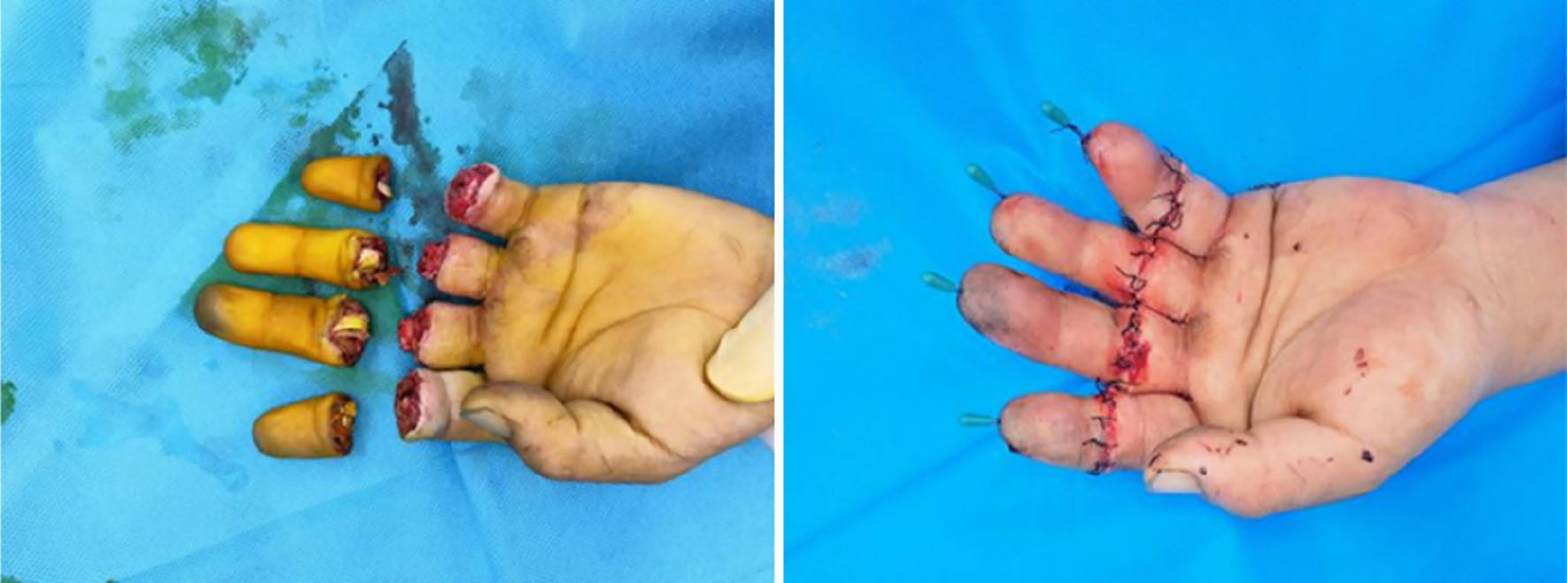
Broken finger (Left) and replantation of broken fingers (Right)

Finger replantation was performed according to the requirements and protocols of “Hand Surgery Operations and Techniques” [23], and the analgesic pump was started 40 min before completion of the operation (Fig. 2).

#### Control group

In the control group, 1 mL of papaverine injection (Shandong Beida Hi-Tech Huatai Pharmaceutical Co., Ltd., State Pharmacopoeia H20052331, 30 mg), 10 mL of tolidansetron hydrochloride injection (Beijing Shuanglu Pharmaceutical Co., Ltd., State Pharmacopoeia H20052460, 5 mg (tolidansetron)), and 100 mL of 0.9% sodium chloride injection were used as self-administered analgesic injections. Patients were placed in a quiet and clean room; their affected limbs were elevated and warmed using a baking lamp, and the skin temperature, skin color, tension, and capillary filling time of the replanted fingers were observed every 1–2 h for 7–10 d after surgery. The patients were advised bed rest and given psychological care; anticoagulation, anti-inflammation, antitussive, and analgesic treatments were also prescribed to the patients.

#### Observation group

The observation group was given clustered care on the basis of the control group. First, an intervention group for the provision of clustered nursing care was established, which consisted of one chief physician, one deputy chief physician, three attending physicians, and one deputy chief nurse practitioner of hand surgery, all of whom had postgraduate degrees and more than 15 years of work experience; one attending physician from the psychology department, who had postgraduate degrees; and five nursing practitioners in charge of the department of Hand, Foot and Ankle Surgery, all of whom had graduate degrees and more than five years of work experience. The chief physician led the intervention group. The members of the cluster intervention group; the directors of the psychology department, gastroenterology department, and orthopedics department; and nursing experts in the hospital discussed and researched together to develop the following clustered nursing intervention plan: (1) Psychological care: Compared with conventional care, in the observation group, a psychological intervention group consisting of one psychologist and two nurses was first set up to actively communicate with patients, understand their psychological status, and patiently enlighten and inspire them. (2) Environmental management: In the observation group, one or two replantation patients were placed in a single ward after surgery, with one escort allowed per bed to reduce visits and personnel movement; the room temperature was controlled at 20–25 °C and the humidity at 50–70%, and the air was disinfected regularly using an air disinfection machine 1–2 times a day for 1 h each to create a good ward environment for the patients. (3) Pain management: Patients were educated at the time of admission about the hazards of pain and the importance of scientific analgesia. Pain medication was administered preoperatively according to the patient pain score to reduce pain. The pain level of patients was assessed using a numerical rating system (NRS) [24] immediately after surgery and 1–3 days after surgery, as suggested by the doctor to understand the pain status of patients. (4) Postural management: After surgery, patients were asked to rest in a flat position and not to get up and move around during the bed rest period. The patient was informed of the importance of resting in a flat position and instructed to alternate between the flat position and healthy side position to prevent complications, such as pressure injury. (5) Urination and defecation care: In the observation group, nursing staff explained, in detail, the hazards of urinary retention and constipation to patients to enhance active compliance with treatment. (6) Blood flow observation: The intervention group trained the members of the observation group about blood flow observation. (7) Form-based management: In the observation group, laboratory and examination results regarding their the mental status, vital signs, urine, stool, blood flow, blood release, pain, special medication, and sleep duration of the reimplanted patients were recorded in detail using the hospital's self-designed microsurgical monitoring record sheet (Table S1), which aims at simplifying nursing case writing. (8) Quality control: Members of the intervention group of the observation group developed a program implementation form based on six aspects of the clustered nursing care program: psychological guidance, environmental management, pain management, blood flow observation, postural management, and constipation care. The implementer marked “yes” or “no” against components of the checklist and signed it after completion.

#### Venous blood collection

Fasting venous blood was collected from both experimental groups on postoperative days 2 and 7; whole blood specimens were placed at 25 °C for 2 h and centrifuged at 1000 × *g* for 15 min at 4 °C. The supernatant was pipetted into 200-μL Eppendorf tubes and stored in a refrigerator at − 20 °C after numbering.

### Observation indexes and assessment criteria

The survival rate of the reimplanted finger: On the 7th day after surgery, the survival rate of reimplanted fingers was compared between the two experimental groups.

Incidence of constipation: The incidence of constipation during the patients' hospitalization was counted separately. Constipation is defined as a change in the normal bowel pattern, a decrease in the number of bowel movements, excessively dry and hard stools, and poor or difficult bowel movements or a feeling of incomplete bowel movements. In this study, the time between bowel movements and the time spent on bowel movements were used as indicators to compare the incidence of constipation between the two groups.

The survival rate of the reimplanted finger: On the 7th day after surgery, the survival rate of the reimplanted finger was compared between the two groups of patients. Determination of the survival of the reimplanted finger [25]: If the capillary filling state of the end of the reimplanted finger was good, the color was red, the finger was fuller and more elastic, and the skin temperature tended to be normal, the finger was deemed to have survived.

Satisfaction: On the 7th postoperative day, our self-developed satisfaction questionnaire (Table S2) was used to investigate the patients’ satisfaction with regards to the supervising physician's skills, the attitude of the responsible nurse, and the functional unit’s services; the physicians' satisfaction with regards to medical and nursing cooperation, medical and nursing communication, work intensity, service attitude, work motivation, blood flow observation, professional knowledge, operation level, medical order execution, and overall evaluation; and the nurses’ satisfaction with regards to work intensity, clarity of content, collective cohesion, reflection of personal value, exertion of subjective initiative, exertion of creativity, nurse manager management, personal improvement, professional development opportunities, and overall evaluation of nurses' satisfaction. The satisfaction surveys for doctors and nurses included 10 items each, with each having 10 points. The total score was of 100 points, and the higher the score, the higher was considered the satisfaction.

Inflammatory factors: Interleukin-1 (IL-1), Interleukin-10 (IL-10) and Tumor necrosis factor-α (TNF-α) were determined by enzyme-linked immunosorbent assay (ELISA), and kits were provided by Wuhan Philosophic Doctor (PhD) Biological Engineering Co.

Vascular crisis: On postoperative day 2 and 7, the reimplantation site was explored by Doppler ultrasound diagnostic instrument (Philips IU-Elte), the resistance index (RI), vascular diameter, mean velocity (Vm), and pulsatility index (PI) were measured. The blood flow parameters *RI* < 0.8, vessel diameter > 0.06 cm, Vm > 5 cm/s and *PI* < 2.0 reflected good circulation status.

Adverse reactions: jaundice, flushing, gastrointestinal discomfort.

ELISA Kit for PI3K (ml360578), AKT (ml601105), mTOR (ml250325) protein expression concentration: After thawing, the samples were centrifuged again and tested according to the kit operating instructions (Enzyme Link Biotechnology Co.). The absorbance of each well was measured sequentially at 450 nm within 15 min after termination. The sample concentration was calculated from the absorbance.

### Statistical analysis

The 110 patients in this study were enrolled, minus the 10 patients who did not meet the inclusion criteria, resulting in 100 patients who met the inclusion requirements. All data were analyzed using the SPSS22.0 statistical software. The data types of the sample experiments were all measures, and the experimental data were expressed as x ± s. If the data in each group were normally distributed, differences between the groups were assessed using multi-sample one-way ANOVA. If the data in each group had a chi-squared distribution, the LSD (last significant digit first) method was used to perform a two-way comparison between groups. If the data in each group did not have a Chi-squared distribution, the Games-Howell method was used to perform a two-way comparison between the groups. If the data in each group were not normally distributed, the multi-sample rank sum test was used. The significance level α was set at 0.05, and the difference between groups was considered statistically significant when *P* < 0.05.

## Results

### Comparison of general information between the two experimental groups

There were 50 patients in the observation group, 25 males and 25 females; their ages ranged from 23 to 58 years, with a mean of 42.25 ± 5.12 years, and their time from injury to hospital admission ranged from 1 to 6 h, with a mean of 3.21 ± 0.54 h. There were 50 patients in the control group, 26 males and 24 females; their ages ranged from 24 to 59 years, with a mean of 42.79 ± 5.06 years, and their time from injury to hospital admission ranged from 1 to 6 h, with a mean of 3.38 ± 0.47 h. The difference in general data between the two groups was not statistically significant (*P* > 0.05), suggesting that the groups were comparable.

### Comparison of pain scores and incidence of constipation between the two experimental groups

The NRS-based pain scores of the two experimental groups in the immediate postoperative period were not significantly different (*P* > 0.05); the pain scores in the observation group were 2.28 ± 0.34, 1.35 ± 0.34, and 0.49 ± 0.12 on days 1, 2, and 3 after surgery, respectively, and were lower than the pain scores in the control group, which were 3.38 ± 0.45, 2.32 ± 0.38, and 1.35 ± 0.24 (*P* < 0.05) on days 1, 2, and 3 after surgery, respectively (Table 1). The incidence of constipation in both groups was found to be 0.73 ± 0.35 d and 7.47 ± 2.12 min shorter in the observation group than in the control group, which were 2.67 ± 0.45 d and 20.45 ± 3.24 min (*P* < 0.05) (Table 1).

**Table 1:** Comparison of pain scores between the two groups

Group	*n*	Pain score				Incidence of constipation	
		Immediate postoperative period	1 d	2 d	3 d	Bowel interval (d)	Defecation time (min)
Observation group	50	1.15 ± 0.23	2.28 ± 0.34**	1.35 ± 0.34**	0.49 ± 0.12**	0.73 ± 0.35**	7.47 ± 2.12**
Control group	50	1.17 ± 0.26	3.38 ± 0.45	2.32 ± 0.38	1.35 ± 0.24	2.67 ± 0.45	20.45 ± 3.24
*t*	/	0.825	6.475	5.347	22.682	4.367	7.513
*P*	/	0.248	0.000	0.000	0.000	0.021	0.000

Compared to the control group, **P < 0.01, n = 3

### Comparison of the survival rate of replanted fingers between the two groups

**Fig. 3:**
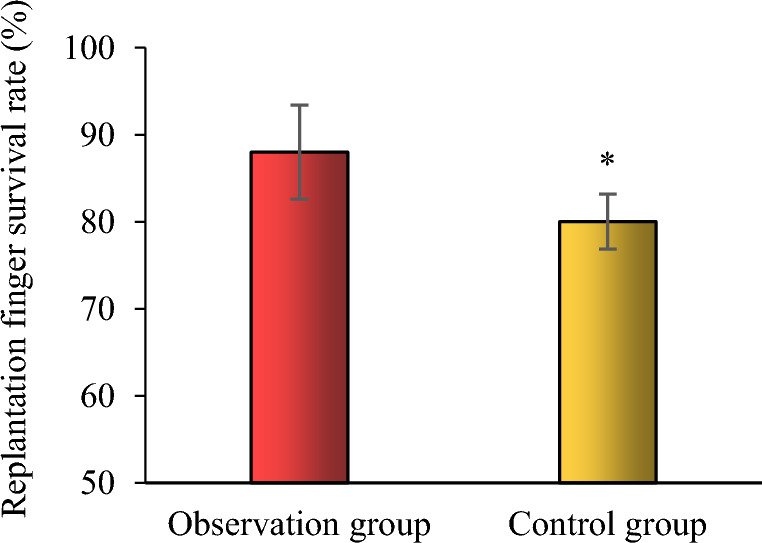
Comparison of the survival rate of replanted fingers between the two groups. Compared to the control group, *P < 0.05, n = 3

The survival rate of replanted fingers in the observation group was 88.00%, which was higher than that of the control group 80.00% (*P* < 0.05) (Fig. 3).

### Comparison of physician, nurse and patient satisfaction between the two experimental groups

The satisfaction scores of doctors, nurses and patients in the observation group were 96.64 ± 5.78, 98.03 ± 6.42 and 98.49 ± 5.63, which were higher than those of the control group 71.46 ± 4.28, 70.84 ± 4.39 and 75.25 ± 6.31 (*P* < 0.05) (Table 2).

**Table 2:** Comparison of physician, nurse and patient satisfaction between the two groups (points)

Group	*n*	Doctors	*n*	Nurses	*n*	Patients
Observation group	8	96.64 ± 5.78**	16	98.03 ± 6.42**	50	98.49 ± 5.63**
Control group	8	71.46 ± 4.28	16	70.84 ± 4.39	50	75.25 ± 6.31
*t*		5.318		6.582		5.859
*P*		0.003		0.001		0.002

Compared to the control group, **P < 0.01, *n* = 3

### Comparison of inflammatory factor indexes between the two groups at each postoperative time point

The differences were not statistically significant (*P* > 0.05) when comparing the inflammatory factor indexes of the two groups on the 2nd postoperative day; IL-1 and TNF-α were lower and IL-10 was higher in the observation group than in the control group on the 7th postoperative day, and the differences were statistically significant (*P* < 0.05) (Table 3).

**Table 3:** Comparison of inflammatory factor indices between the two groups at each postoperative time point

Group	IL-1		IL-10		TNF-α	
	2 d	7 d	2 d	7 d	2 d	7 d
Observation group (n = 50)	31.35 ± 12.49	27.41 ± 11.68**	35.79 ± 12.93	32.48 ± 10.15**	325.13 ± 30.15	313.79 ± 31.12**
Control group (n = 50)	31.28 ± 12.53	38.97 ± 13.46	35.74 ± 12.89	26.21 ± 7.36	324.85 ± 30.26	417.83 ± 43.28
*t*	0.028	6.212	0.019	5.848	0.045	12.653
*P*	0.979	0.000	0.984	0.002	0.967	0.000

IL-1, Interleukin-1; IL-10, Interleukin-10; TNF-α, Tumor necrosis factor-α; Compared to the control group, **P < 0.01, *n* = 3

### Comparison of vascular crisis parameters between the two groups at each postoperative time point

The differences were not statistically significant (*P* > 0.05) when the vascular crisis parameters were compared between the two groups on the 2nd postoperative day; the RI and PI of the observation group on the 7th postoperative day were lower than those of the control group, and the vascular diameter and Vm were higher than those of the control group, and the differences were statistically significant (*P* < 0.05) (Table 4).

**Table 4:** Comparison of vascular crisis parameters at each postoperative time point between the two groups

Group	Vascular diameter (cm)		Vm (cm/s)		RI		PI	
	2 d	7 d	2 d	7 d	2 d	7 d	2 d	7 d
Observation group (n = 50)	0.04 ± 0.01	0.14 ± 0.03**	4.58 ± 0.37	7.56 ± 2.09**	0.91 ± 0.03	0.69 ± 0.08**	2.42 ± 0.34	1.73 ± 0.20**
Control group (n = 50)	0.04 ± 0.01	0.06 ± 0.02	4.68 ± 0.40	5.27 ± 0.88	0.89 ± 0.03	0.83 ± 0.06	2.44 ± 0.29	2.15 ± 0.25
*t*	0.000	16.054	0.513	6.545	1.783	10.642	0.452	8.629
*P*	1.000	0.000	0.619	0.000	0.078	0.000	0.657	0.000

Vm, Average blood flow rate; RI, Blood Flow Resistance Force Index; PI, Arterial Pulsatility Index. Compared to the control group, **P < 0.01, *n* = 3

### Comparison of the occurrence of adverse reactions in the two groups

**Fig. 4:**
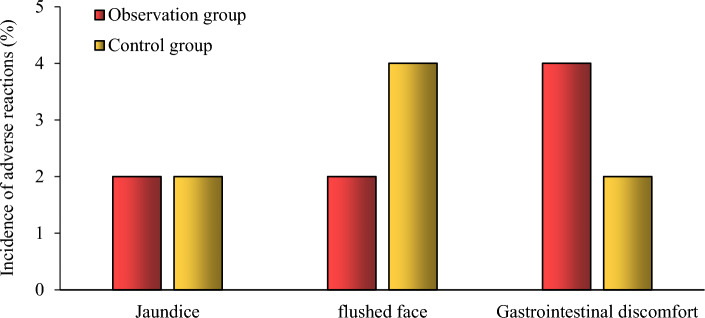
Comparison of the occurrence of adverse reactions between the two groups

The incidence of adverse reactions jaundice, flushing and gastrointestinal discomfort were 1 (2.0%), 1 (2.0%) and 2 (4.0%) in the observation group and 1 (2.0%), 2 (4.0%) and 1 (2.0%) in the control group, respectively, and the difference was not statistically significant when compared with the incidence of adverse reactions in the two groups (*P* > 0.05) (Fig. 4).

### Comparison of phosphatidylinositol 3-kinase (PI3K), protein kinase B (AKT), and mammalian target of rapamycin (mTOR) protein concentrations between the two experimental groups at each postoperative time point

On postoperative day 2, the differences in PI3K, AKT, and mTOR protein concentrations between the two experimental groups were not statistically significant (*P* > 0.05). On postoperative day 7, the PI3K, AKT, and mTOR protein concentrations in the observation group were all significantly higher than those in the control group (*P* < 0.05) (Table 5).

**Table 5:** Comparison of PI3K, AKT, and mTOR protein concentrations at each postoperative time point between the two groups

Group	PI3K (pg/mL)		AKT (ng/mL)		mTOR (ng/mL)	
	2 d	7 d	2 d	7 d	2 d	7 d
Observation group (*n* = 50)	432.67 ± 13.48	457.98 ± 14.24**	3.38 ± 0.16	3.62 ± 0.17**	33.68 ± 1.34	34.95 ± 1.45**
Control group (*n* = 50)	431.79 ± 12.82	552.80 ± 15.89	3.42 ± 0.19	4.27 ± 0.24	33.57 ± 1.65	46.48 ± 1.85
*t*	0.034	10.482	0.019	8.938	0.047	11.723
*P*	0.963	0.000	0.984	0.002	0.924	0.000

PI3K, phosphatidylinositide3-kinases; AKT, serine-threonine kinase; mTOR, mammalian target of rapamycin. Compared to the control group, **P < 0.01, *n* = 3

## Discussion

Replantation of severed fingers is a treatment method aimed at restoring blood circulation by microscopically attaching severed nerves, tendons, blood vessels, and other tissues back at their original position. Pain is an independent risk factor for the development of vascular crisis after finger replantation. This is mainly because post-traumatic pain stimulates the body to release a variety of injury-related factors, such as prostaglandins and 5-hydroxytryptamine, leading to strong vasoconstriction [26, 27]. Therefore, reducing postoperative pain is crucial to ensure the survival of reimplanted fingers. In the present study, the NRS-based pain scores in the immediate postoperative period in the two experimental groups were not significantly different (*P* > 0.05); the pain scores in the observation group were 2.28 ± 0.34, 1.35 ± 0.34, and 0.49 ± 0.12 on postoperative days 1, 2, and 3, respectively, and were lower than the pain scores in the control group, which were 3.38 ± 0.45, 2.32 ± 0.38, and 1.35 ± 0.24 on postoperative days 1, 2, and 3, respectively (*P* < 0.05). The pain levels gradually decreased over time. These findings indicate that clustered care can significantly relieve postoperative pain in patients who have undergone replantation. Postoperative restriction of movement for a long time after finger replantation, along with discomfort with defecation in bed, leads to changes in the defecation pattern, reduces frequency of defecation, and difficulty in defecation. In the present study, patients in the control group had a longer defecation interval and took a longer time to defecate. The incidence of constipation in both groups was 0.73 ± 0.35 d and 7.47 ± 2.12 min, which were shorter than those in the control group 2.67 ± 0.45 d and 20.45 ± 3.24 min, indicating that clustered care can effectively shorten the interval and time of defecation and reduce the incidence of constipation in patients after finger replantation. The quality of care after finger replantation is sometimes directly related to the success or failure of the replantation surgery. Therefore, the choice of nursing model is crucial. In the present study, the survival rate of replanted fingers in the observation group was 88.00% and was higher than that in the control group, which was 80.00% (*P* < 0.05).

The present study also confirms that the strategy involving cluster management of controllable factors of vascular crisis can effectively prevent vascular crisis after the replantation of severed fingers. The satisfaction scores of physicians, nurses, and patients in the observation group were 96.64 ± 5.78, 98.03 ± 6.42, and 98.49 ± 5.63, respectively, and were higher than those in the control group, which were 71.46 ± 4.28, 70.84 ± 4.39, and 75.25 ± 6.31, respectively (*P* < 0.05). The reasons for this may be that the cluster nursing intervention increased opportunities for patient-nurse communication, enhanced the patients' sense of being cared for and respected, provided catharsis for negative emotions, reduced the incidence of pain and constipation, enhanced patient comfort, increased the survival rate of the reimplanted finger, saved the patients from physical disability, reflected the humane and systematic characteristics of nursing services, and improved the patients' medical experience. Therefore, in future clinical work, nursing staff should continuously improve their scientific research ability as well as enrich centralized nursing measures. A systematic, standardized, and centralized nursing process can guarantee the nursing effect, improve the quality of nursing care, and reduce patients’ pain.

As a commonly used vasodilator, papaverine inhibits phosphodiesterase activity in vascular smooth muscle cells and the inward flow of calcium across vascular smooth muscle cell membranes, dilating peripheral vasculature, organ vessels, and bronchial and digestive smooth muscles; it has thus been widely used for the prevention and treatment of vasospasm after finger amputation [28, 29]. With the introduction of a new method for papaverine injection, the side effects of papaverine have decreased; however, papaverine-induced vascular inflammation is still common [30]. The present study showed that the concentrations of IL-1, TNF-α, RI, and PI were lower in the observation group than those in the control group on postoperative day 7 (*P* < 0.05), and the concentration of IL-10, vascular vessel diameter, and Vm were higher than in the control group that those in the observation group (*P* < 0.05); the incidences of adverse reactions were similar between the two groups (*P* > 0.05).

The PI3K/Akt/mTOR signaling pathway is an important metabolic signal transduction pathway that is involved in a variety of physiological and pathological activities in vivo, including cell proliferation, differentiation, growth, metabolism, migration, apoptosis, protein synthesis, sugar transport, vesicle transport, and cytoskeletal structure development [31]. It is the main component of PI3K/Akt/mTOR signaling pathway [32]. AKT is activated by PI3K through phosphorylation [33, 34]. mTOR and AKT are both serine/threonine kinases, and three isoforms of AKT protein genes have been identified in mammals. mTOR undergoes two-way activation through PI3K/AKT. In the present study, there was no statistically significant difference between the PI3K, AKT, and mTOR protein concentrations on postoperative day 2 between the two experimental groups (*P* > 0.05). The PI3K, AKT, and mTOR protein concentrations on postoperative day 7 were significantly higher in the observation group than those in the control group (*P* < 0.05). These results indicated that clustered care intervention could significantly enhance the concentrations of PI3K, AKT, and mTOR in the peripheral blood of patients after finger replantation and thereby stimulate vascular neovascularization by activating the PI3K/AKT/mTOR pathway. This could promote the repair and healing of recanalized blood vessels after surgery, improving the survival rate of fingers after replantation.

Clustered nursing interventions have achieved good results in the prevention of vascular crisis after finger replantation and are worthy of clinical promotion. However, there are many limitations in this study. First, the number of cases included in the trial was relatively low due to a small study duration; further validation with a large sample size is needed. Second, there is a certain amount of human error involved in performing the collection and statistical analysis of patient data: additional studies to confirm our findings should thus be conducted.

## Conclusions

In conclusion, the findings of the present study suggest that clustered nursing intervention combined with papaverine injection can reduce the incidence of postoperative pain, constipation, and vascular crisis; improve the survival rate of reimplanted fingers; improve serum inflammatory factor levels; and inhibit vascular inflammation; regulate vascular crisis parameters in patients who have undergone surgery for replantation of severed fingers. Moreover, this care model can improve the satisfaction of physicians, nurses, and patients, and is therefore worthy of clinical application.

### Supplementary Information

Below is the link to the electronic supplementary material.

Supplementary file1 (DOCX 21 kb)

## Data Availability

Data will be made available on request.
