# Supplementary material for: Effect of PI3K/AKT/mTOR signaling pathway-based clustered nursing care combined with papaverine injection on vascular inflammation and vascular crisis after replantation of severed fingers
Source: Mol Cell Biochem. 2023 Jul 25;479(6):1525–34. doi: 10.1007/s11010-023-04796-y (PMC11224086; doi:10.1007/s11010-023-04796-y)
Supplement: Supplementary file 1 — Supplementary file1 (DOCX 21 kb) [file 11010_2023_4796_MOESM1_ESM.docx]

Table S1 Microsurgery test record sheet

| Time | 25 ℃ | Healthy side skin temperature | Flat temperature | | | | | Color | | | | | Red and white reaction | | | | | Fullness | | | | | Swelling | | | | | Peripheral arterial pulsation | | | | | Special case records |
| --- | --- | --- | --- | --- | --- | --- | --- | --- | --- | --- | --- | --- | --- | --- | --- | --- | --- | --- | --- | --- | --- | --- | --- | --- | --- | --- | --- | --- | --- | --- | --- | --- | --- |
|  |  |  |  |  |  |  |  |  |  |  |  |  |  |  |  |  |  |  |  |  |  |  |  |  |  |  |  |  |  |  |  |  | Day shift signature: |
| 8: 00 |  |  |  |  |  |  |  |  |  |  |  |  |  |  |  |  |  |  |  |  |  |  |  |  |  |  |  |  |  |  |  |  |  |
| 9: 00 |  |  |  |  |  |  |  |  |  |  |  |  |  |  |  |  |  |  |  |  |  |  |  |  |  |  |  |  |  |  |  |  |  |
| 10: 00 |  |  |  |  |  |  |  |  |  |  |  |  |  |  |  |  |  |  |  |  |  |  |  |  |  |  |  |  |  |  |  |  |  |
| 11: 00 |  |  |  |  |  |  |  |  |  |  |  |  |  |  |  |  |  |  |  |  |  |  |  |  |  |  |  |  |  |  |  |  |  |
| 12: 00 |  |  |  |  |  |  |  |  |  |  |  |  |  |  |  |  |  |  |  |  |  |  |  |  |  |  |  |  |  |  |  |  |  |
| 13: 00 |  |  |  |  |  |  |  |  |  |  |  |  |  |  |  |  |  |  |  |  |  |  |  |  |  |  |  |  |  |  |  |  |  |
| 14: 00 |  |  |  |  |  |  |  |  |  |  |  |  |  |  |  |  |  |  |  |  |  |  |  |  |  |  |  |  |  |  |  |  |  |
| 15: 00 |  |  |  |  |  |  |  |  |  |  |  |  |  |  |  |  |  |  |  |  |  |  |  |  |  |  |  |  |  |  |  |  |  |
| 16: 00 |  |  |  |  |  |  |  |  |  |  |  |  |  |  |  |  |  |  |  |  |  |  |  |  |  |  |  |  |  |  |  |  |  |
| 17: 00 |  |  |  |  |  |  |  |  |  |  |  |  |  |  |  |  |  |  |  |  |  |  |  |  |  |  |  |  |  |  |  |  |  |
| 18: 00 |  |  |  |  |  |  |  |  |  |  |  |  |  |  |  |  |  |  |  |  |  |  |  |  |  |  |  |  |  |  |  |  |  |
| 19: 00 |  |  |  |  |  |  |  |  |  |  |  |  |  |  |  |  |  |  |  |  |  |  |  |  |  |  |  |  |  |  |  |  |  |
| 20: 00 |  |  |  |  |  |  |  |  |  |  |  |  |  |  |  |  |  |  |  |  |  |  |  |  |  |  |  |  |  |  |  |  | Night shift signature: |
| 21: 00 |  |  |  |  |  |  |  |  |  |  |  |  |  |  |  |  |  |  |  |  |  |  |  |  |  |  |  |  |  |  |  |  |  |
| 22: 00 |  |  |  |  |  |  |  |  |  |  |  |  |  |  |  |  |  |  |  |  |  |  |  |  |  |  |  |  |  |  |  |  |  |
| 23: 00 |  |  |  |  |  |  |  |  |  |  |  |  |  |  |  |  |  |  |  |  |  |  |  |  |  |  |  |  |  |  |  |  |  |
| 00: 00 |  |  |  |  |  |  |  |  |  |  |  |  |  |  |  |  |  |  |  |  |  |  |  |  |  |  |  |  |  |  |  |  |  |
| 1: 00 |  |  |  |  |  |  |  |  |  |  |  |  |  |  |  |  |  |  |  |  |  |  |  |  |  |  |  |  |  |  |  |  |  |
| 2: 00 |  |  |  |  |  |  |  |  |  |  |  |  |  |  |  |  |  |  |  |  |  |  |  |  |  |  |  |  |  |  |  |  |  |
| 3: 00 |  |  |  |  |  |  |  |  |  |  |  |  |  |  |  |  |  |  |  |  |  |  |  |  |  |  |  |  |  |  |  |  |  |
| 4: 00 |  |  |  |  |  |  |  |  |  |  |  |  |  |  |  |  |  |  |  |  |  |  |  |  |  |  |  |  |  |  |  |  |  |
| 5: 00 |  |  |  |  |  |  |  |  |  |  |  |  |  |  |  |  |  |  |  |  |  |  |  |  |  |  |  |  |  |  |  |  |  |
| 6: 00 |  |  |  |  |  |  |  |  |  |  |  |  |  |  |  |  |  |  |  |  |  |  |  |  |  |  |  |  |  |  |  |  |  |
| 7: 00 |  |  |  |  |  |  |  |  |  |  |  |  |  |  |  |  |  |  |  |  |  |  |  |  |  |  |  |  |  |  |  |  |  |
| Notes：I. Color: 0, normal; 1, slightly purple; 2, dark purple; 3, slightly white; 4, pale; 5, gray; 6, blackish. II. Red-white reaction: 0, normal; 1, rapid; 2, slow; 3, not obvious; 4, not present. III. Terminal arterial pulsation: Yes√; No ×. IV. Fullness: 0, normal; 1, under full; 2, dry. V. Swelling: 0, no swelling; 1, swollen skin lines existed; 2, swelling obvious skin lines disappeared; 3, moderate swelling with blisters. | | | | | | | | | | | | | | | | | | | | | | | | | | | | | | | | | |

Table S2 Inpatient satisfaction questionnaire

| Dear Patients:  Hello, "Your satisfaction, is our pursuit, your recovery, is our wish. In order to further strengthen the construction work of the practice and improve the quality of medical services, we have set up the "Inpatient Questionnaire" and invite you to participate in the evaluation. Please evaluate the following issues separately, and there are three levels of evaluation, namely, very satisfied, satisfied, unsatisfied. There are three levels of evaluation, namely, very satisfied, satisfied and unsatisfied, and issues that are not covered cannot be evaluated. Thank you for your cooperation. | | | | |
| --- | --- | --- | --- | --- |
| Please mark "√" for the following survey content as you see fit. | | | | |
| Content | Very satisfied | Satisfied | Dissatisfaction | Not experienced |
| 1. How do you rate the section in general? |  |  |  |  |
| 1. Are you satisfied with the service attitude of the supervising physician? |  |  |  |  |
| 1. How skilled do you think the supervising physician is? |  |  |  |  |
| 1. Are you satisfied with the service attitude of the charge nurse? |  |  |  |  |
| 1. How skilled do you think the charge nurse is? |  |  |  |  |
| 1. Do you think the charge nurse provided you with timely assistance when you were in trouble? |  |  |  |  |
| 1. Do you think the charge nurse makes frequent ward rounds? |  |  |  |  |
| 1. Are you satisfied with the environmental hygiene of the ward? |  |  |  |  |
| 1. Are you satisfied with the hospital's fees: the inquiry service provided, the status of the fee list? |  |  |  |  |
| 1. Are you satisfied with the hospital's diet? |  |  |  |  |
| 1. Are you satisfied with the services of the Laboratory Department? |  |  |  |  |
| 1. Are you satisfied with the services of the Pharmacy Department and Ultrasound Room? |  |  |  |  |
| 1. Are you satisfied with the services of the Pathology Department and the Blood Transfusion Department? |  |  |  |  |
| 1. Are you satisfied with the logistic support service of our hospital? |  |  |  |  |
| 1. Are you satisfied with the integrity of the medical staff? |  |  |  |  |
| 1. What is your overall opinion of the hospital? |  |  |  |  |
| 1. Did the medical staff provide you with health education and advice during the consultation? |  |  |  |  |
| 1. Are you satisfied with the health education guidance provided by the medical staff? |  |  |  |  |
| 1. Are you satisfied with the services of the radiology department and Computed Tomography room? |  |  |  |  |
| 1. Do you think that the signs in the hospital are clear and eye-catching? |  |  |  |  |
| Notes: Calculation method: 5 points for "very satisfied", 3 points for "satisfied", 0 points for "dissatisfied", and the total score was calculated by excluding the item of "not experienced". | | | | |
